# Understanding Creutzfeldt-Jackob disease from a viewpoint of amyloidogenic evolvability

**DOI:** 10.1080/19336896.2020.1761514

**Published:** 2020-05-07

**Authors:** Makoto Hashimoto, Gilbert Ho, Yoshiki Takamatsu, Ryoko Wada, Shuei Sugama, Masaaki Waragai, Eliezer Masliah, Takato Takenouchi

**Affiliations:** a Laboratory for Parkinson's Disease, Tokyo Metropolitan Institute of Medical Science, Setagaya-ku, Japan; b PCND Neuroscience Research Institute, Poway, CA, USA; c Department of Physiology, Nippon Medical School, Tokyo, Japan; d Division of Neurosciences, National Institute on Aging, National Institutes of Health, Bethesda, MD, USA; e Institute of Agrobiological Sciences, National Agriculture and Food Research Organization, Tsukuba, Japan

**Keywords:** Creutzfeldt-Jackob (CJD), sporadic CJD, genetic CJD, acquired CJD, evolvability, amyloidogenic proteins (APs), prion protein (PrP), α-synuclein (αS), antagonistic pleiotropy

## Abstract

Creutzfeldt-Jackob disease (CJD), the most common human prion disorder, is frequently accompanied by ageing-associated neurodegenerative conditions, such as Alzheimer’s disease and Parkinson’s disease. Although cross-seeding of amyloidogenic proteins (APs), including amyloid β and α-synuclein, may be critical in the co-morbidity of neurodegenerative disorders, the direct interaction of APs with prion protein (PrP), the central molecule involved in the pathogenesis of CJD, is unlikely. Currently, the nature of this biological interaction and its significance remain obscure. In this context, the objective of the present study is to discuss such interactions from the perspective of amyloidogenic evolvability, a putative function of APs. Hypothetically, both hereditary- and sporadic CJD might be attributed to the role of PrP in evolvability against multiple stressors, such as physical stresses relevant to concussions, which might be manifest through the antagonistic pleiotropy mechanism in ageing. Furthermore, accumulating evidence suggests that PrP- and other APs evolvability may negatively regulate each other. Provided that increased APs evolvability might be beneficial for acquired CJD in young adults, a dose-reduction of α-synuclein, a natural inhibitor of αS aggregation, might be therapeutically effective in upregulating APs evolvability. Collectively, a better understanding of amyloidogenic evolvability may lead to the development of novel therapies for CJD.

## 1. Introduction

Creutzfeldt-Jackob disease (CJD) is a fatal degenerative brain disorder that is associated with various progressive symptoms, including dementia, involuntary movements, blindness, and coma [1–3]. Biochemically, it was found that the neurotoxic conversion of the prion protein (PrP), a conserved GPI-anchored membrane protein, into the misfolded forms of PrP may play a central role in the pathogenesis of CJD [4]. Histopathologically, the CJD brain is characterized by extensive spongiform changes in grey matter, accompanied by gliosis, neuropil rarefaction, neuron loss, and deposition of misfolded PrP [5]. CJD is clinically divided into three main categories. Whereas both sporadic- and hereditary CJD forms are chronic neurodegenerative diseases in ageing, the acquired CJD form occurs as an infectious condition of younger adults, caused by exposure to infected tissues containing an infectious form of PrP (PrP^sc^) [4]. Despite extensive efforts, CJD remains without an effective disease-modifying or disease-preventing therapy.

Recently, there has been a great interest in the co-morbidity of ageing-associated neurodegenerative diseases [6], including CJD which frequently co-occurs with ageing-associated neurodegenerative diseases such as Alzheimer’s disease (AD) and Parkinson’s disease (PD) [4]. Although it is generally believed that the co-morbidity of neurodegenerative conditions might be attributed to cross-seeding of amyloidogenic proteins (APs) [6], current results, however, suggest that PrP may not directly interact with other APs, such as amyloid β (Aβ) and α-synuclein (αS) [7,8].

Given that the relationship between sporadic- and hereditary CJD is similar to those between sporadic- and hereditary ageing-associated neurodegenerative disorders, we propose that PrP might also be related to and be influenced mechanistically by evolvability. In this context, the main objective of the present study is to explore the functions and interactions of PrP from the standpoint of amyloidogenic evolvability, a putative function of APs [9]. According to our view [9], the possible role of PrP in evolvability in reproductive stage might be manifest as neurodegeneration in ageing through the antagonistic pleiotropy mechanism. Furthermore, accumulating evidence suggests that PrP and other APs evolvability may negatively regulate each other. Finally, it is expected that novel therapeutic strategies could be developed against acquired CJD, where no therapy exists, based on the evolvability hypothesis.

## 2. PrP and neurodegenerative disease

**Figure 1.**
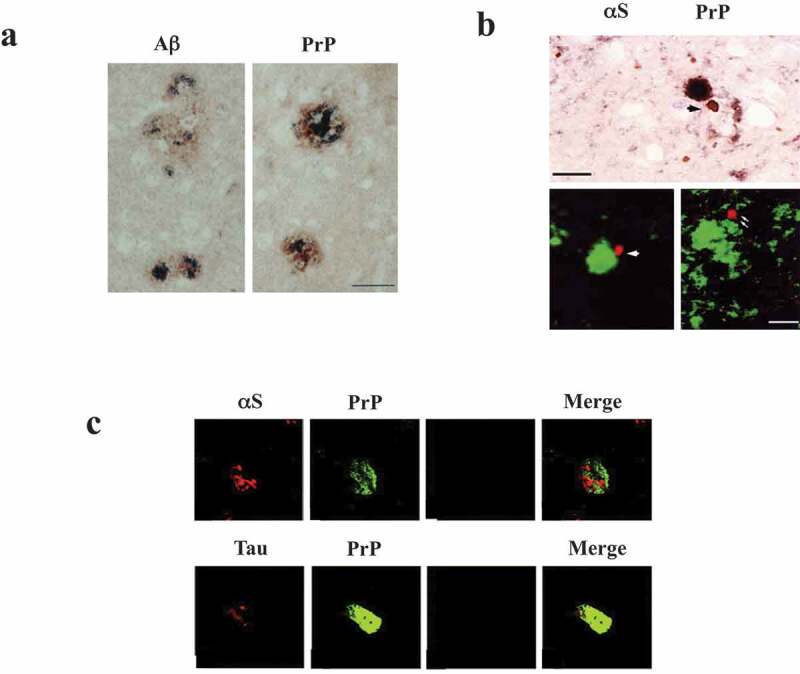
Co-occurrence of CJD with other neurodegenerative diseases. (a). Immunohistochemistry of Co-localization of PrP (brown precipitate) and Aβ (dark blue precipitate) in senile plaques in AD. Expression of PrP was observed, although it was unlikely that PrP directly bound with Aβ. Bar 50 μm. (b). Double labelling of αS and PrP. Upper; immunohistochemistry of experimental scrapie of hamsters. DAB substrate for αS (brown) and DAB-nickel substrate for PrP (dark brown) were used. Lower; double immunofluorescence by confocal microscopy revealed PrP- and αS-immunoreactivities in the plaques were not strictly co-localized. αS immunolabeling was found either isolated (arrow) or in areas of PrP granular deposits (double arrow) or close to PrP plaques (arrowhead). DAB diaminobenzidine, FITC fluorescein isothio-cyanate, Cy3 cyanin 3. Bar 15 μm (c). Double immunofluorescence showed that PrP was not overlapped with either αS nor tau in fully developed inclusions in AD, PD and DLB. Reprinted from Ferrer et al. [7] (a), Haik et al. [8] (b) and Kovacs et al. [10] (c) with permission.

Similar to ageing-associated neurodegenerative disorders, a number of histopathological studies have observed that PrP pathology is frequently co-localized with those of other APs. For instance, PrP expression was observed within senile plaques in AD, although it was unlikely that PrP directly bound to Aβ [7] (Figure 1(a)). Furthermore, αS-immunoreactive deposits were identified in the central nervous system of various prion diseases, including sporadic, iatrogenic and new variant CJD, while the immunoreactivity of PrP and those of APs were not strictly co-localized particularly in the plaques, in experimental scrapie of hamsters [8] (Figure 1(b)). Moreover, double immunofluorescence showed focal overlapping of PrP^C^ with tau and with αS in early, but not in fully developed inclusions, in various neurological diseases, including AD, PD and dementia with Lewy bodies (DLB) (Figure 1(c)) [10]. Thus, it has been suggested that PrP aggregation might occur independently of other APs. Consistent with this notion, characterization of AD/age-related tauopathy co-pathology in CJD showed independent pathogenic mechanisms, suggesting no cross-seeding between misfolded Aβ and PrP [11]. Considering that cross-seeding of APs might be critical in promoting neurodegenerative diseases during ageing [6,12], PrP might play a distinct role in the pathogenesis of neurodegenerative disorders compared to other APs.

## 3. Evolvability of PrP

Given the shared quality of protein aggregation among PrP and other APs, it is reasonable to speculate that PrP also might be involved in evolvability. Indeed, APs evolvability was proposed based on the evolvability of yeast prion [9].

### 3.1. The concept of amyloidogenic evolvability

To date, the function of neurodegeneration-relevant amyloidogenic proteins (APs), such as Aβ and αS, in the human brain is essentially unknown. In this regard, we recently proposed that evolvability might be one physiological function of APs [9,13]. More specifically, the diverse β-sheet structures of protofibrillar APs forms might confer resistance against multiple stressors, namely hormesis, in parental brains, which may be transmitted to offspring through germ cells [9,13]. By virtue of the stress information derived from parental brains, an offspring’s brain can better cope with forthcoming stressors to avoid developmental/early life disorders [14]. On the contrary, neurodegeneration may manifest in parental brains through the antagonistic pleiotropy mechanism in ageing [13].

### 3.2. PrP evolvability

Similar to other APs, protofibrillar PrP with diverse β-sheet structures are characterized as ‘intrinsically disordered proteins’ [15]. By virtue of such unique structures, it is possible that PrP might correspond to the diverse stressors [16]. Considering that the prion infectivity of PrP is much stronger than those of other APs, and transmission may be a critical process in evolvability [13], it is reasonable to predict that PrP may be stronger than those of other APs. Although the details of PrP evolvability remain unclear, it is worth noting that plasma soluble PrP is implicated as a potential biomarker for sport-related concussions [17], suggesting that PrP might be involved in evolvability against physiological stressors. It would be intriguing to determine in an animal study if the transgenerational effects of the resistance against concussions might depend on the expression levels of PrP. Notably, the transgenerational (mother-to-offspring) transmission of PrP was previously evaluated using tg mouse model expressing bovine PrP [18]; however, the concept of amyloidogenic evolvability was not described. As long as we know, our model is the first to refer to the association of PrP with evolvability.

## 4. Reciprocal relationship of APs- with PrP evolvability

It is likely that the interaction of PrP with other APs might be not simply a matter of cross-seeding between aggregate-prone proteins. However, the underlying biological mechanisms and significance of this currently remain obscure. Given that neurodegenerative diseases might be attributed to evolvability in which multiple APs might cooperate in a well-regulated system [12], we speculate that PrP might be important as a regulator of the evolvability by other APs in addition to its role in evolvability against physiological stressors.

### 4.1. APs fibrils decrease PrP evolvability

**Figure 2.**
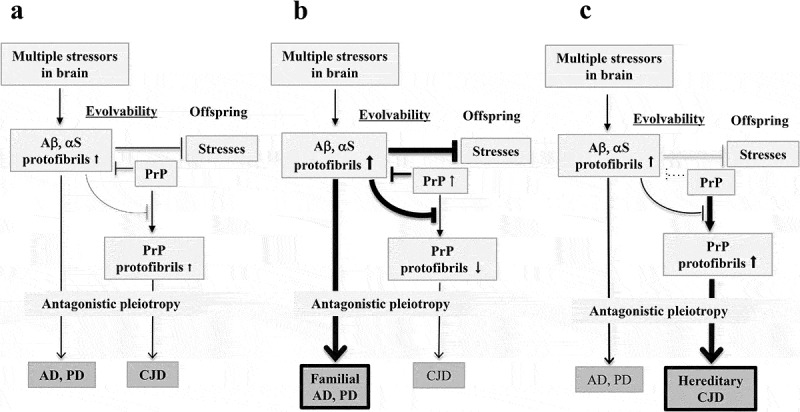
Schematic of the interaction of PrP with other APs in evolvability and in neurodegenerative diseases. (a). Under normal conditions, monomeric PrP acts as a receptor for APs oligomers to enter into cells to facilitate the cell-to-cell spreading of APs [19,20]. Furthermore, PrP monomer may regulate expression of Aβ through α-secretase cleavage of amyloid precursor protein [23]. On the other hand, APs oligomers were shown to block the replication of PrP [21], leading to the upregulation of the PrP monomer:PrP oligomer ratio. Collectively, PrP may act as a positive regulator of APs evolvability. Although the interaction of PrP with other APs in evolvability results in neurodegenerative disorders in ageing, the risk is elevated. (b). When the aggregation of APs is predominant compared to that of PrP, AP oligomers may inhibit the conversion of monomeric PrP to protofibrils. Consequently, neurodegenerative disorders, such as AD and PD, may predominate over CJD. This might occur with missense mutations of APs and other susceptible genes. (c). Conversely, if PrP aggregation is predominant compared to those of APs, CJD may predominate over other neurodegenerative diseases, including AD and PD.

Notably, fibrillar APs, including Aβ and αS, employ soluble monomeric PrP as a neuronal receptor for entry into cells, facilitating cell-to-cell spreading of APs [19,20]. Furthermore, it was shown that the replication of PrP was blocked by fibrillar APs [21]. Mechanistically, it was suggested that αS might suppress the nucleation step of PrP amyloidogenesis presumably through direct interaction between αS and PrP intermediates [22]. Thus, it is predicted that AP fibril formation might suppress PrP evolvability (Figure 2(a)), and this view is in agreement with the observation that the accumulation of AP, such as amyloid deposits, in CJD patients is accompanied by a longer disease course [21], which may be a clue towards a therapeutic opportunity.

### 4.2. PrP decreases APs evolvability

Conversely, PrP was shown to suppress α-secretase cleavage of the amyloid precursor protein, resulting in decreased Aβ expression [23], suggesting that PrP might decrease APs evolvability. Supposing that the effect of monomeric PrP is abrogated by aggregation, this feedback mechanism might be interpreted as a regulatory system through protein aggregation (Figure 2(a)). Thus, AP fibril formation might be suppressed by monomeric PrP and vice versa, suggesting that PrP evolvability and other APs evolvability may negatively regulate each other.

## 5. A ‘superior-inferior’ relationship between CJD and other neurodegenerative diseases

It has been suggested that co-morbidity of CJD with other neurodegenerative diseases reveals heterogeneous patterns in disease progression. Some cases are characterized by the predominance of CJD over other neurodegenerative diseases, whereas others are not [24–28]. Although the mechanism is poorly understood, the evolvability viewpoint might provide some insight into this important phenomenon.

### 5.1. Dominance of other neurodegenerative diseases over CJD

Several studies previously described that the duration of CJD disease progression might be more protracted when accompanied by other neurodegenerative diseases, such as AD and PD [24–28]. For instance, one case with predominant AD pathology superimposed on CJD showed a significantly longer duration of illness (~5 years) [26], which contrasts with two other cases showing pathological predominance of CJD over AD, revealed a shorter duration of illness (around 1 year) [25,28].

According to our concept of evolvability, such interactions between APs and PrP may be mechanistically explainable in terms of the regulation of evolvability. In this regard, if the aggregation of APs is predominant over that of PrP, then oligomeric APs may inhibit the conversion of monomeric PrP to protofibrils. Consequently, neurodegenerative disorders, such as AD and PD, may be the predominant clinical phenotype over CJD (Figure 2(b)). Indeed, one mechanism of such disease predominance relationship may be missense mutations in neurodegeneration-related APs leading to increased AP aggregation and inhibition of PrP fibrillization. Alternatively, the risk of sporadic neurodegenerative diseases might be increased through various susceptibility genes, such as apolipoprotein E (APOE), which might be perhaps also negatively influencing PrP aggregation. Indeed, the APOE alleles were previously described as major susceptibility factors for CJD [29].

### 5.2. Dominance of CJD over other neurodegenerative diseases

Conversely, in patients with both PrP and other AP co-pathology, if PrP aggregation predominates over those of other APs, CJD may be phenotypically predominant over other neurodegenerative diseases, such as AD and PD (Figure 2(c)). Yet, provided the low frequency of hereditary CJD (10 ~ 15%) containing a disease-causing PrP mutation compared to sporadic CJD (~85%) [1], such predominance of CJD pathology may be infrequent. Still, it is possible that beyond missense mutations, PrP polymorphisms might promote such disease predominance of CJD. Alternatively, AP-associated neurodegenerative diseases might also harbour protective genes, which might reduce the aggregative properties of APs relative to PrP, also leading to PrP predominance.

## 6. Evolvability and acquired CJD: therapeutic implications

In addition to sporadic and hereditary CJD that represent chronic neurodegenerative disorders of ageing, the third form of CJD, namely acquired CJD, occurs as an infectious disease preferentially in young adults. Acquired CJD is caused by exposure to the tissues with the infected from various sources [4]. Although acquired CJD is rare (~1%), the transmission of a fatal and untreatable neurological disorder has had major implications for public health and public policy.

### 6.1. Involvement of evolvability in acquired CJD?

**Figure 3.**
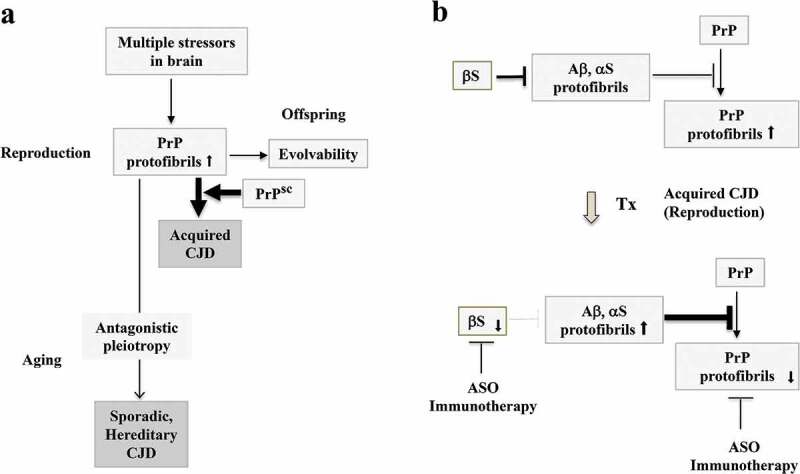
Evolvability and acquired CJD from a viewpoint of evolvability. (a). Schematic illustration of the central role of evolvability in the pathophysiology of CJD. During reproduction, PrP protofibrils formed in response to multiple stressors in the parental brain might be transgenerationally transmitted to confer resistance against the multiple stressors in offspring’s brain, otherwise designated as the concept of evolvability. Thus, evolvability of PrP may be beneficial in evolution. In case of sporadic and hereditary CJD, neurodegeneration might be manifest through the antagonistic pleiotropy mechanism in ageing. During the reproductive time of life, the levels of PrP protofibrils might be increased for evolvability, and under these circumstances, exposure to the infectious PrP may readily lead to acquired CJD. (b). Therapeutic strategy against acquired CJD during reproduction from a viewpoint of evolvability. One current therapeutic strategy against CJD is the dose-reduction of PrP by various methods, such as ASO and immunization to decrease the formation of toxic amyloid fibrils of PrP, and delay the progression of CJD. Yet, the efficacy of such a therapy has yet to be improved. Since it is possible that the dose-reduction of αS by either ASO or immunotherapy may result in upregulation of fibrillar APs, including αS and Aβ, which leads to PrP suppression. Thus, a combined therapy strategy of targeting both PrP and αS might be more effective.

Given that acquired CJD behaves as an infectious disorder, it might appear to be unrelated to evolvability, a phenomenon which is beneficial to offspring. We propose, however, that amyloidogenic evolvability may indeed be of relevance to acquired CJD as well. According to the concept of amyloidogenic evolvability, it is assumed that the expression level of PrP protofibrils in individuals at reproductive age may already be significantly elevated in order to respond to multiple brain stressors that would be transmitted to offspring as evolvability. As described above, plasma soluble PrP is upregulated by sport-related concussions [17], suggesting that PrP might be involved in evolvability against physical stressors. Such a view may be reasonable considering that resistance against concussions may be critical during the delivery of newborn babies. Thus, it is predicted that PrP evolvability might be elevated in young adults. Under these conditions, exposure to exogenous infectious PrP protofibrils may readily cross the species barrier, resulting in the propagation of abnormal endogenous PrP, and subsequently, rapidly progressing acquired CJD (Figure 3(a)). Thus, evolvability of PrP may be mechanistically important in all CJD forms, including acquired CJD.

## 7. A proposed therapeutic strategy against acquired CJD

Presuming that PrP evolvability might play a central role in the pathogenesis of all forms of CJD, an evolvability-based therapeutic strategy might, therefore, be engineered against CJD based on the potential mechanisms discussed above.

### 7.1. Interfering with PrP protein aggregation

Similar to other neurodegenerative conditions, a dose-reduction of PrP might be sufficiently effective to reduce the formation of toxic amyloid fibrils of PrP and delay CJD progression. Certainly, the promising results of both active and passive immunization of PrP have been reported in animal models of prion diseases [30]. Furthermore, antisense oligonucleotide (ASO) of PrP was shown to extend the survival of prion-infected mice [31]. Collectively, these findings emphasize the therapeutic potential of PrP dose-reduction for treating patients with CJD (Figure 3(b)).

### 7.2. An evolvability-based CJD therapeutic strategy

Considering that the pathogenesis of CJD develops much more rapidly compared to those of other ageing-associated neurodegenerative disorders, it is likely that interfering with protein aggregation in CJD should be very effective. For this purpose, an evolvability-based therapeutic strategy might be combined with anti-protein aggregation methods. As described, it is interesting that the propagation of PrP is negatively regulated by αS fibrils [21]; however, we predict that such an effect is effective only during reproductive life and not during older age. As such, it is thought that upregulation of APs, including Aβ and αS, might result in suppression of PrP replication, leading to amelioration of acquired CJD, but not other CJD forms during ageing (Figure 3(b)). With this in mind, exogenous αS might be administered therapeutically by either injection or infusion, yet, other adverse effects might limit its utility. Alternatively, since αS inhibits αS aggregation [32] and binds with Aβ [33], suppressing αS expression by various methods, including antisense oligonucleotides against αS mRNA and immunotherapy against the αS protein, might also effectively increase aggregation of APs, leading to a suppression of PrP evolvability (Figure 3). To extend this further, a combined therapy of both dose reduction in PrP and anti-αS approaches might be an even more efficacious therapeutic strategy against acquired CJD. Yet, such therapy approaches that target upregulation of αS or Aβ may be ‘a double-edged sword’, in which, on the one hand, they might be effective against acquired CJD during reproductive life, but during ageing, the therapy may instead promote the development of age-associated, AP-associated neurodegenerative conditions.

## 8. Concluding Remarks

As per our discussion, we predict that the evolvability of PrP might be centrally situated in the pathogenic mechanism of all subtypes of CJD. As long as the evolvability of PrP is an evolutionarily beneficial trait, CJD might persist against the pressures of natural selection. In particular, our hypothesis may explain why acquired CJD, a devastating disorder usually found in young adults during reproductive life (being evolutionarily disadvantageous), has emerged and persisted during evolution. Thus, since the risk of acquired CJD, however low, is ever persistent in the population, it may be necessary to devise novel therapies against this condition, for which no therapy is presently available.

Since AP protofibrils, including that of Aβ and αS, which are formed for evolvability, might suppress the replication of PrP, this might be important from a therapeutic standpoint. Accordingly, since upregulation of αS aggregation is beneficial during the reproductive time of life, downregulation of αS, the endogenous inhibitor of αS aggregation, might be therapeutically beneficial for acquired CJD. Collectively, a better understanding of the concept of amyloidogenic evolvability may provide novel insights into a more comprehensive mechanism-based therapy approach against CJD.
